# Supplementary figures and images for: Epithelial polarization in the 3D matrix requires MST3 signaling to regulate ZO-1 position
Source: PLoS One. 2023 May 8;18(5):e0285217. doi: 10.1371/journal.pone.0285217 (PMC10166550; doi:10.1371/journal.pone.0285217)

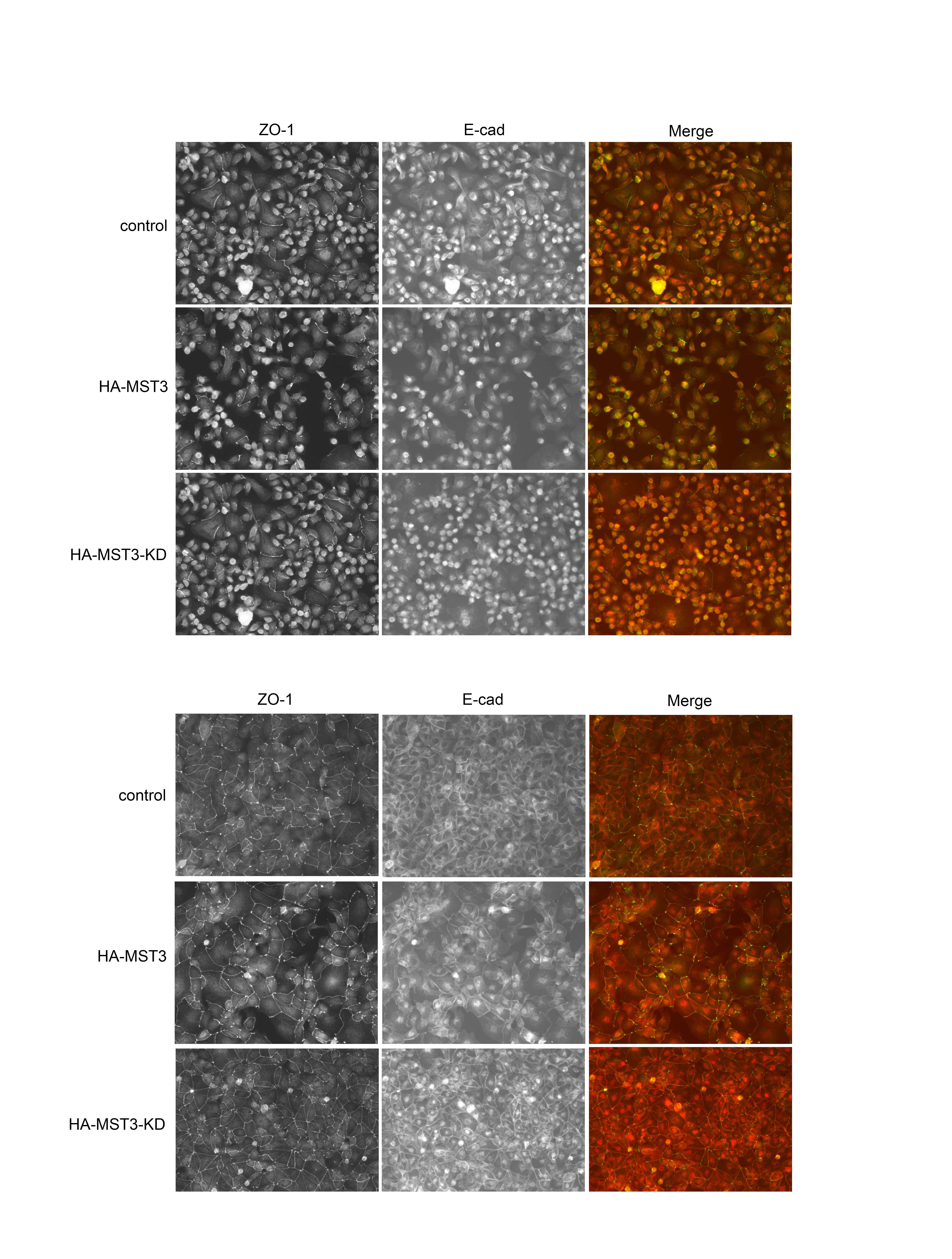

Supplement: S1 Fig — Representative images of 3 × 10^5^ control, HA-MST3, and HA-MST3 KD cells grown on collagen-coated coverslips with large field. After 72 h, the medium was changed to medium that was depleted of calcium for 24 h. Cells were fixed at (A) 1 h and (B) 4 h after the readdition of calcium and stained for ZO-1 (green) and E-cad (red) with X20. (JPG) [file pone.0285217.s001.jpg]
